# Recombinant activated factor VII (Novo7^®^) in patients with ventricular assist devices: Case report and review of the current literature

**DOI:** 10.1186/1749-8090-2-47

**Published:** 2007-10-26

**Authors:** Daniel Heise, Anselm Bräuer, Michael Quintel

**Affiliations:** 1 University Hospital Goettingen, Department of Anesthesiology, Emergency and Intensive Care Medicine, Goettingen, Germany

## Abstract

Postoperative bleeding might become a serious problem in the management of cardiac surgical patients, with marked medical and economic impact. In these life-threatening situations, massive haemorrhage represents frequently a combination of surgical and coagulopathic bleeding. Surgical bleeding results from a definite source at the operation site and can be corrected using surgical standard techniques. Acute coagulopathies, in contrast, result from impaired thrombin formation, and require optimized therapeutical strategies. Effective pharmacological treatment will be complicated by the presence of ventricular assist devices (VAD), which necessarily imply effective anticoagulation.

In episodes of uncontrolled coagulopathic bleeding, the application of recombinant activated factor VII (rFVIIa) as a effective haemostatic agent has become more and more popular. However, only very few data are available on its use in patients with VAD in place.

We researched the PubMed-database for case reports about the use of rFVIIa in patients with VAD and summarized them. In addition, we report a case from our hospital. In all cases cessation of bleeding without any thrombembolic complications could be achieved. In cases of uncontrollable, non-surgical bleeding rFVIIa seems to be a therapeutical option even for patients with VAD.

## Introduction

Over the last decade, the impact of ventricular assist devices (VAD) in the treatment of end-stage cardiac failure has been increasingly recognized. Next to elective "bridge-to-transplant"-implantation, VAD are frequently used during cardiac surgery procedures if weaning from cardiopulmonary bypass is refractory due to poor cardiac performance. Out of 115 VAD inserted during a six-year-period at a major tertiary referral centre in New York for example, 63% were non-elective [1]. While using these devices, however, the risk of major non-surgical bleeding is high, especially during and early after cardiac surgery, as VAD require an efficient anticoagulation because of their thrombogenic surface [2].

Massive postoperative haemorrhage is frequently a combination of surgical and coagulopathic bleeding. Surgical bleeding results from a definite source at the operation site and can be corrected using traditional surgical techniques (e.g., ligation of vessels, cauterization, etc.). In contrast, acute coagulopathies result from impaired thrombin formation, develop early after surgical trauma, and thus may seriously complicate these life-threatening situations. For several years, rFVIIa (NovoSeven^®^) has been approved for haemophilic patients with inhibitors. However, in episodes of uncontrolled postoperative bleeding, the off-label use of rFVIIa as a potentially effective, but cost-intensive haemostatic agent has been rapidly expanding. rFVIIa links with tissue factor, which is released from injured tissue, and then activates factor IX and X, respectively. Subsequently, the formation of fibrin is initiated. In addition, supra-normal concentrations of FVIIa lead to an increased thrombin synthesis in platelets as well as to an activation of cellular haemostasis [3]. Especially in patients with VAD and uncontrolled non-surgical bleeding, the application of rFVIIa is a therapeutical challenge as thrombembolic complications may lead to severe dysfunctions of these devices.

In this article, we report the application of rFVIIa in a patient with a biventricular assist device (BIVAD) in order to treat massive non-surgical bleeding, and present a review on the current literature.

## Case report

A 22 year old man with a history of congenital dilatative cardiomyopathy (left ventricular ejection fraction < 20%) was admitted to the medical ICU after acute cardiac decompensation. Despite the application of inotropics at maximum doses (incluiding levosimendan), it was not possible to restore an adequate cardiac performance, and subsequently, even mechanical cardiopulmonary resuscitation had to be started. As an ultima ratio procedure, emergency thoracotomy was immediately performed, the patient was heparinized (300 I.U./kg) and rapidly connected to extracorporal circulation. After implantation of a left ventricular assist device (LVAD) and reperfusion, weaning from cardiopulmonary bypass failed due to an acute right ventricular failure. Consequently, a right-ventricular assist device (RVAD) was additionally implanted, and extracorporal circulation could be stopped without complications.

After this emergency procedure, the patient was transferred to the surgical ICU in stable haemodynamic conditions. However, the patient was continuously and massively bleeding, with a blood loss of > 500 mL per 15 minutes. It was necessary transfuse 10 units of red blood cells (RBC), 7 units of frozen plasma (FFP) and 10 units of platelet concentrate (PC), and to substitute 3000 I.U. of prothrombin complex (PPSB), 3000 I.U. of antithrombin and 6 g of fibrinogen immediately after arrival on the ICU. Despite these efforts, massive blood loss persisted and a re-thoracotomy was performed at the bedside. During this procedure, surgical reasons for the haemorrhage at the site of surgery could be definitely excluded. After this procedure again 10 units of RBC, 14 units of FFP, 7500 I.U. of PPSB and 7000 I.U. of protamine were given without any significant effect. Laboratory values showed a clearly impaired coagulation (PT 44%, PTT 88 sec., ACT 229 sec., fibrinogen 124 g/dl, platelets 59 × 10^3^/μL). In situation, we decided to apply rFVIIa, and after a single dose of 120 μg/kg, bleeding stopped within a few minutes. Fortunately, the function of the VAD remained completely unimpaired.

In the further course, however, the patient developed acute renal failure, and increasing doses of norepinephrine and finally vasopressin were necessary in order to maintain an adequate perfusion pressure. Liver enzymes massively increased, and furthermore, extracorporal membrane oxygenation had to be initiated due to the rapid development of severe lung failure. 9 days after the implantation of the VAD a fixed bilateral mydriasis became apparent, and clinical examination revealed the complete absence of brain stem reflexes. Subsequently, a transcranial Doppler examination was performed, which revealed a complete stop of cerebral perfusion. This examination was repeated after twelve hours and confirmed the absence of cerebral blood flow. Based on these findings, the decision was made to stop any therapeutical efforts, and VADs were switched off leading to an immediate circulatory arrest.

## Review of the literature

To our knowledge, four cases have been published so far referring to the therapy with rFVIIa in patients with VAD.

Flynn and co-workers report on a 44 years old male patient who suffered from an ischemic cardiomyopathy and received a LVAD because of chronic deterioration of his cardiac performance. [4] As several weaning attempts failed, the temporary VAD was switched to a permanent device ("artificial heart") using cardiopulmonary bypass. Despite the fact that activated clotting time (ACT) after reversal of heparin was <150 sec, a diffuse haemorrhage exceeding 1500 mL/h from all chest tubes occurred. After the substitution of 11 units of FFP, 7 units of PC and PPSB (in an unknown dose) the bleeding still continued. Hence, rFVIIa was administered in a single dose of 90 μg/kg, and blood loss immediately decreased below 100 mL/h. The following postoperative course was uncomplicated, the patient was listed for heart transplantation and discharged from hospital with the permanent assist device.

In a case report of Kogan et al., a 48 years old male patient underwent heart transplantation due to severe ischemic cardiomyopathy [5]. After aortic declamping, an episode of a electromechanical dissociation occurred without a perceptible reason. Adequate left ventricular performance could only be restored applying high doses of catecholamines (25 μg/kg/min dobutamine, 0,8 μg/kg/min epinephrine and 4,2 μg/kg/min norepinephrine, respectively) and inserting an intraaortic counterpulsation pump. As the right ventricle remained akinetic, it was necessary to implant a RVAD. After a total bypass time of more than 9 hours, a diffuse and massive haemorrhage became evident and persisted despite the administration of 20 units of RBC, 15 units of FFP and 16 units of PC. In this situation, laboratory investigations revealed an impaired coagulation (INR 2,86, PTT > 120 sec., fibrinogen 115 mg/dl, platelets 78.000/μl). After application of two doses of rFVIIa (2 × 35 μg/kg, in an interval of 60 minutes) the bleeding suspended, and the patient could be transferred to the surgical ICU. In the following, right ventricular function slowly recovered, and the application of nitric oxide (NO) finally led to a complete recompensation within 24 hours. The patient was discharged from hospital on the 19. postoperative day.

In another publication, Potapov and co-workers report on a 57 years old woman with acute myocarditis and severe cardiogenic shock [6]. Despite of a high-dose therapy with catecholamines (1 μg/kg/min epinephrine, 7 μg/kg/min dobutamine, 0,7 μg/kg/min norepinephrine), low cardiac output persisted. To maintain adequate systemic perfusion a biventricular assist device (BIVAD) was implanted. Postoperatively, a massive blood loss of more than 1000 mL/h required the transfusion of 30 units of RBC. In an attempt to optimize coagulation, a total of 56 units of FFP, 4 units of PC, 2000 I.U. of PPSB, 28 μg desmopressine, protamine and aprotinine (unknown doses) were administered. Nevertheless, the bleeding persisted despite a apparently normal coagulation status (INR 1,39, PTT 42,5 sec., fibrinogen 212 g/dl, AT 83%). 12 hours postoperatively, a single dose of 120 μg/kg rFVIIa was given, and blood loss immediately decreased to < 500 mL/h. Two hours later, a second dose of 60 μg/kg rFVIIa (and two units of PC) were applied, and the bleeding rate further decreased to <100 mL/h. The function of the BIVAD was not affected, especially no thromboembolic complications were encountered. However, the patient's outcome remains unclear in this case report.

Zietkiewicz et al. report on a 34 years old man undergoing elective replacement of a mitral valve prosthesis [7]. Two operations – a mitral valve anuloplasty as well as a tricuspid anuloplasty with the initial replacement of the mitral valve – preceded this procedure. Postoperatively, the patient developed a severe cardiogenic shock refractory to high doses of inotropes (no further data applicable). In order to relief the impaired left ventricle and to maintain adequate organ perfusion, a LVAD was implanted. Within the first 6 postoperative hours, diffuse bleedings of >1000 mL/h from chest tubes, wounds and from the nose occurred. RBC, FFP, PC and tranexamic acid were administered (doses not applicable). Nevertheless, massive blood loss persisted in spite of the fact that laboratory parameters for coagulation were nearly unaffected (INR 1,61, AT 62%, fibrinogen 271 mg/dL, platelets 89.000/μl). In the following, the patient received 20 μg/kg rFVIIa, and during the next two hours, the haemorrhage decreased to 25 mL/h. However, as blood loss increased again 4 hours later, a a second dose of 30 μg/kg rFVIIa was given which led to complete cessation of the bleeding. Again, the function of the LVAD was not impaired at all. Unfortunately, 7 days later, an acute tension pneumothorax caused an acute cardiac decompensation. Despite the immediate initiation of chest drains, cardiac performance was refractory to pharmacological treatment, and the patient died.

In our case, where finally clinical and ultrasound doppler signs of brain death were obsereved we can, even if it seems unlikely, not definitievly exclude that a thromobembolic complication caused massive ischemic cerebral infarction followed by massive brain edema, resulting in a stop of cerebral perfusion.

## Discussion

Transfusion of autologous blood products is not only cost-intensive, but implies also the risk of several serious adverse events. Many clinical studies clearly document the direct inter-relation between the number of transfused units and the incidence of nosocomial infections, the duration of hospital stay, the number of ventilator days, mortality, the incidence of acute renal failure and cardiac complications, respectively [8-11]. Thus, the perioperative reduction of blood products appears to be a very important strategy which should start with the preoperative identification of patients at risk of massive blood loss. In cardiac surgery, for example, the risk of bleeding is significantly elevated when anticoagulation is performed with hirudine [12], in the presence of septic endocarditis [13] or hepatic disorders [14], after preoperative administration of platelet aggregation inhibitors [15], re-operations [16] and emergency procedures, respectively [17]. In this respect, the adequate management of patients undergoing VAD implantation represents a particular challenge to the physicians involved, as permanent cannulation of large vessels as well as the obligatory anticoagulation both tremendously increase the risk of postoperative haemorrhage [2].

Even if the treatment of non-haemophilic patients with rFVIIa represents an "off label" use, this agent is known to be an effective haemostatic agent in uncontrolled haemorrhage caused by a wide spectrum of clinical scenarios [18]. Especially after cardiac surgery, it is a well-known fact that even massive bleedings following anticoagulation with lepirudine may sufficiently be treated with rFVIIa [19]. The effect of rFVIIa on plasmatic coagulation derives from its interaction with tissue factor. Thus, the pro-coagulatory effect is predominantly located in regions where tissues or vessels are injured [3]. This intriguing characteristic might be an explanation for the relatively low incidence of thrombembolic events (1–2%) after the use of rFVIIa [20]. Marson and co-workers even treated a patient with rFVIIa who preoperatively suffered from repeated deep venous thromboses and who therefore had placed a filter within the inferior caval vein. However, no thrombembolic complications were encountered [21]. Recently, O'Connell et al. published a detailed analysis of 431 „adverse event reports” in conjunction with the use of rFVIIa. [22]. 168 of these voluntary declarations referred to thrombembolic complications – „assist devices” (in a broader sense, including dialysis shunts and even endotracheal tubes etc.) were affected in only 10 cases. In this respect, thrombembolic complications in patients with VAD were not explicitly mentioned, and thus seem to be very rare. Nevertheless, the authors point out that these reports were voluntary, and thus, their analysis probably underestimates the absolute number of complications after application of rFVIIa.

In 2005, Von Heymann and co-workers published their experiences with 26 patients who were successfully treated with rFVIIa after cardiac surgery without any thrombembolic complications. Interestingly, three of these patients were in need of a VAD.

The reviewed cases clearly suggest that the exact timing of the treatment with rFVIIa plays an important role. In this respect, an untimely administration seems to be as disadvantageous as a delayed therapy, as additional costs and an increased risk of further blood transfusions may be caused. Using pharmaco-economic criteria, Loudon and Smith determined the optimal time point for the application of rFVIIa after transfusion of 14 units of red blood cells [23]. Restrictively, they point out that this is the result of a theoretical analysis which implies the prompt cessation of bleedings directly after therapy with rFVIIa. However, another analysis of 50 patients treated with rFVIIa concludes that delayed application of rFVIIa worsens the patient's prognosis [24].

As mentioned above, in O'Connell's analysis of 431 voluntarily reported complications after the use of rFVIIa, only 10 concerned thrombembolic episodes associated with „assist devices” [22]. In spite of the fact that these data suggest that thrombembolism in VAD patients is a very rare complication after the use of rFVIIa, it may cause life-threatening dysfunctions, and hence, the seriousness of such events should not be underestimated. Nevertheless, in 36 of 50 lethal complications, the most probable cause of death was embolism, for example in pulmonary or coronary arteries. Thus, independent of the presence or absence of VAD, these data suggest that the application of rFVIIa generally implies the risk of serious thrombembolic complications.

## Conclusion

In this article, we report about the successful application of rFVIIa in a patient with biventricular assist device (BIVAD) in order to treat massive non-surgical bleeding, and give a brief review on the current literature. We conclude that in a uncontrolled non-surgical haemorrhage, the administration of rFVIIa seems to be a promising therapeutic option even in patients with VAD, especially when „conventional” treatment (substitution of coagulation factors or platelets, desmopressine, antifibrinolytics) is not effective. In this respect, the exact timing for starting the treatment with rFVIIa is important in order to minimize the risks of mass transfusion. Nevertheless, rFVIIa therapy in patients with VAD requires meticulous monitoring, with special regard to thrombembolic events. In addition, the repeated application of small doses (e.g. 30–40 μg/kg), if necessary, might, under these circumstances, be an effective and cost saving procedure.

## Competing interests

The author(s) declare that they have no competing interests.

**Table 1:** Summary of previously published case reports about the use of rFVIIa in patients with ventricular assist devices

	Flynn et al., [4]	Kogan et al., [5]	Potapov et al., [6]	Zietkiewicz et al., [7]
Type of VAD	LVAD	RVAD	BIVAD	LVAD
Blood loss before rFVIIa	1500 mL/h	Massive bleeding	1000 mL/h	1000 mL/h
Therapy before administration of rFVIIa	11 FFP 7 PC. PPSB*	15 FFP 16 PC. (20 units RBC)	56 FFP 4 PC. 2000 i.U. PPSB 28 μg desmopressine protamine* aprotinine* (30 units RBC)	FFP* PC* tranexamic acid* (RBC)*
Laboratory values before administration of rFVIIa	not reported	INR: 2,86 PTT: > 120 sec. Fib.: 115 mg/dl PLT.: 78.000/μL	INR: 1,39 PTT: 42,5 sec. Fib.: 212 mg/dl AT: 83% PLT.: 54.800/μL	INR: 1,61 AT: 62% Fib.: 271 mg/dL PLTr.: 89.000/μL
**Dose of rFVIIa**	**90 μg/kg**	**2 × 35 μg/kg**	**120 + 60 μg/kg**	**20 + 30 μg/kg**
Blood loss after rFVIIa	< 100 mL/h	0	< 100 mL/h	< 15 mL/h
Laboratory values after administration of rFVIIa	not reported	INR: 1,3 PTT: 34 sec. Fib.: 127 mg/dL PLT.: 88.000/μl	INR: 1,19 PTT: 47 sec. Fib.: 144 mg/dL AT: 52% PLT.: 75.700/μL	INR: 0,89
Clinical course/outcome	Discharged from hospital with permanent LVAD, listed for heart transplantation	Recompensation, explantation of RVAD, discharged from hospital	No thromboembolic complications. Outcome not reported.	No thromboembolic complications. Lethal tension pneumothorax 7 days after implantation of VAD.

*: Dosage not applicable
